# Students' Online Burnout, Fatigue Situations and Associated Factors: A Cross‐Sectional Study

**DOI:** 10.1002/nop2.70750

**Published:** 2026-08-19

**Authors:** Papatya Karakurt, Meryem Firat, Şeyda Yetginler Memiş

**Affiliations:** ^1^ Fundamentals of Nursing Division, Department of Nursing, Faculty of Health Sciences Erzincan Binali Yıldırım University Erzincan Turkey; ^2^ Division of Mental Health and Psychiatric Nursing, Department of Nursing, Faculty of Health Sciences Erzincan Binali Yıldırım University Erzincan Turkey; ^3^ Division of Mental Health and Psychiatric Nursing, Department of Nursing, Health Sciences İnstitute Atatürk University Erzurum Turkey

**Keywords:** burnout, distance learning, health sciences education, mental fatigue, nursing, Zoom

## Abstract

**Aim:**

This study was planned to determine online burnout, fatigue states of students and the associated factors.

**Design:**

This study was designed as a cross‐sectional type of research. The study is of a quantitative type.

**Methods:**

The universe of this study consisted of all 890 students studying in the Department of Nursing and Nutrition Dietetics at the Faculty of Health Sciences in the spring semester of the 2023–2024 academic year at a university in the east of Türkiye. The sample of the study consisted of 570 students studying in the 2nd, 3rd and 4th grades. The ‘Introductory Information Form’ was used to collect data, and the ‘Zoom Burnout and Fatigue Scale’ was used to determine the students' online burnout and fatigue status. Percentage, mean, standard deviation, *t*‐test in independent groups, variance (ANOVA) and Pearson correlation analysis were used in the analysis of data.

**Results:**

The mean age of the students included in the study was determined to be 21.77 ± 2.01; 40.9% were sophomores. It was determined that the students received 2.86 ± 1.07 points from the total scale. The maximum score obtained from the scale is 5. It was determined that there was a significant difference between the mean scores of the scale and its sub‐dimensions according to the difficulty of studying with distance education compared with studying with face‐to‐face education, difficulty in accessing information sources with distance education and difficulty in using information technologies (*p* < 0.05).

**Conclusions:**

As a result, students are showing average levels of the Zoom Burnout and Fatigue. In line with these findings, it can be suggested that when delivering theoretical courses via distance education, active learning methods should be used, students' access to course materials should be facilitated and their participation should be supported or encouraged.

**Relevance to Clinical Practice:**

Since nursing and dietetics are hands‐on profession, practices must take place in a hospital setting and face‐to‐face. Online education should be used as a supplementary tool. Students should apply the online education they receive to their practice.

**Patient or Public Contribution:**

Participants provided data but were not involved in the study's design, outcome measures or conduct.

## 1 Introduction

Online teaching methods differ from traditional lecture methods, and students often face online video meeting fatigue (Toney et al. 2021). The time spent in front of a computer and screen has led to a rapidly growing problem known as Zoom fatigue or burnout, named after the popular video conferencing software. This fatigue, also known as virtual fatigue or Zoom fatigue, affects video conference participants. This condition, which causes Zoom fatigue and burnout in individuals due to participating in video conferences, is a general feeling of mental fatigue and exhaustion caused by video conferencing (Ateş and Kanık 2022).

Burnout is defined as a condition characterized by mental fatigue and detachment from work or stress accumulation that the body can no longer handle (Al‐Zain and Abdulsalam 2022). In the academic context, the adaptation of teaching to the online method has allowed students to continue their studies while maintaining social distance, which has also affected their mental health (Usher et al. 2020). However, in the online method, learning was self‐directed, irregular, and students had to learn under pressure (Velarde‐García et al. 2021). Students experiencing burnout may be due to the high work demands imposed on them by their families or teachers. Fatigue occurs due to a feeling of inefficiency associated with negative beliefs about educational competencies or opportunities (Tomaszek and Muchacka‐Cymerman 2022). This period of online classes is a stressful period (Majrashi et al. 2021), and these processes cause both burnout and fatigue in students. In a study, stress and mental health of nursing students, in addition to the perceived level of support and education, partially explain academic burnout during this period, as higher levels of academic burnout were reported by 3rd‐ and 4th‐year students (Sveinsdóttir et al. 2021). A study conducted in Türkiye showed that demographic factors such as gender, class and department can determine the burnout level of students (Korkmaz et al. 2020). In another study, it was determined that students experienced difficulties in three dimensions: technical, educational and social during the distance education process (Türkmen et al. 2021). With the rapid advancement of technology, online platforms are inevitably used for delivering lessons and preparing materials for assignments. This research aimed to determine the levels of online fatigue and burnout among students, enabling nurses to develop strategies to reduce or prevent these issues.

Distance education, which was a technological necessity during the pandemic, continues to be used as an alternative and/or complement to traditional face‐to‐face education after the pandemic. Digitalization in education, which has accelerated as a revolution during the pandemic, can strengthen the academic interests of students who are familiar with technology and even addicted to it (Ali 2020). After the pandemic process experienced in our country and the earthquake cantered in Kahramanmaraş on 6 February 2023, the distance education process started at all levels of education (Yıldız and Karagözoğlu 2024). Zoom fatigue arises from specific factors such as cognitive overload, limited nonverbal communication cues, constant self‐monitoring (awareness of being on camera) and physical inactivity, all stemming from prolonged screen time in a digital environment; whereas classic academic burnout is a chronic syndrome more closely associated with long‐term workload, role conflict, emotional labour and organizational pressures. Zoom fatigue is noteworthy as a situational and technology‐specific form of burnout that can develop more quickly, whereas academic burnout represents a broader construct encompassing emotional exhaustion, depersonalization and decreased personal accomplishment that accumulate over time. Especially in regions where earthquakes occurred, students' access to the internet has become limited. Due to the earthquake, most students experienced problems with distance education. During this process, it was inevitable for students to experience stress, fatigue and burnout, especially due to not being able to go to practice areas. Due to both the pandemic and the earthquake experienced in our country, students continuing their education with online education increase their anxiety about their professions, increase their reluctance to lessons, creates fatigue, exhaustion and pressure on them. It is inevitable for students of the nursing and nutrition dietetics department to experience more uncertainty as they have to practice the courses they take in hospitals and various institutions. All these situations have caused uncertainty among students, because the fact that students are not informed about sudden decisions leads them to pessimism, causing them to experience burnout and fatigue. Today, the reflection of digital developments in education and training is inevitable. In this process, students need to be aware of digital developments by using technological tools. This research aimed to determine the extent to which digitalization in nursing education affects the levels of fatigue and burnout among students and to provide data for the formation of educational policies aimed at preventing these issues.

The increasing use of distance education during the pandemic is unsuitable for the practical aspects of nursing and dietetics. Students who interact directly with patients, communicate with them one‐on‐one, and empathize with them during practical training cannot do so through distance education. Therefore, distance education leads to fatigue and burnout among students. This research was planned to determine the levels of burnout and fatigue experienced by students due to online education.

This study was planned to determine online burnout, fatigue states of students and the associated factors.

This research sought answers to the following questions:

1. What are the online burnout and fatigue levels of students?
2. What are the factors associated the online burnout and fatigue levels of students?

## 2 Methods

### 2.1 Research Design

This research is a cross‐sectional type of research. The study is of a quantitative type.

### 2.2 Participants and Settings

The population of the study consisted of all 890 students enrolled in the Departments of Nursing and Nutrition and Dietetics at the Faculty of Health Sciences during the spring semester of the 2023–2024 academic year at a university in eastern Türkiye. First‐year students (*n* = 196) were excluded because they did not receive online education during the spring semester of the 2022–2023 academic year due to the earthquakes centred in Kahramanmaraş on 6 February 2023. Therefore, the accessible population of the study consisted of 694 students. The minimum sample size was calculated as 195 students using the sample size formula for a known population, with a 95% confidence interval and a 5% margin of error. A convenience sampling method was used because the study was conducted within a limited time frame and depended on the accessibility and voluntary participation of students who were available during the data collection period. However, this non‐probability sampling method may limit the generalizability of the findings and increase the risk of selection bias.

The study was completed with 570 students who voluntarily agreed to participate and were present during the data collection process. Students who were absent or unwilling to participate at the time of data collection were not included in the study. Inclusion criteria included being 18 years of age or older, being enrolled as a 2nd‐, 3rd‐ or 4th‐year student, having experienced online education during the relevant academic period, and voluntarily agreeing to participate in the study. As the study employed a cross‐sectional design, the findings reflect associations between variables and do not allow for causal inferences. The study reached 82.1% of the population. Data were collected using the convenience sampling method. It was reported using the STrengthening the Reporting of OBservational studies in Epidemiology (STROBE) guidelines (Vandenbroucke et al. 2021).

### 2.3 Data Collection Tools

Data were collected between March and April 2024, the beginning of the spring semester of the 2023–2024 academic year, when there were no course evaluations. The ‘Information Form’ prepared by the researchers will be used to collect the data, and the ‘Zoom Burnout and Fatigue Scale’ will be used to determine the online burnout and fatigue states of the students.

#### 2.3.1 Information Form

This form includes questions about students' age, gender, class status, opinions about online education, etc.

#### 2.3.2 Zoom Burnout and Fatigue Scale

The Zoom Burnout and Fatigue Scale was developed by Fauville et al. (2021). The scale measures feelings of exhaustion and fatigue resulting from participating in videoconferences. The scale is a 5‐point Likert scale (1‐not at all, 5‐a lot). The scale was developed on university students. The Turkish validity and reliability study of the scale was conducted by Sezer and Akça (2023). As a result of the validity and reliability analyses of the scale, the scale consists of 15 items and 5 sub‐dimensions. It is calculated by dividing the average total score of the scale by the number of items in the scale. The mean scores of the subscales are calculated by dividing the total score by the number of items in the subscale (Sezer and Akça 2023). In this study, the Cronbach alpha coefficient of the scale was determined to be 0.96.

### 2.4 Data Sources/Collection

Data collection forms to be used in the research were sent to students via Google form from their social media accounts. Data collection took approximately 3–5 min.

### 2.5 Data Analysis

The data obtained as a result of the research was analyzed in a computer environment using the statistical program SPSS for Windows 25.0. Descriptive statistics, frequencies, percentages, means and standard deviations were used to analyze the data. The normality of the data was calculated using the ‘Kurtosis’ and ‘Skewness’ coefficients (±2). Inferential statistics were also used to test the normal distribution of continuous variables and homogeneity of variances. The *t*‐test was used to report the comparison of the means of the two main dependent variables (Zoom Burnout and Fatigue Scale) with the independent variables (demographic characteristics: gender, department, questions including opinions on distance learning, etc.). ANOVA and LSD post hoc analyses were used to compare the means of the dependent variables with three or more groups (class status, high school graduation, place of residence, perception of family income and number of siblings). The Pearson Correlation analysis was used to compare the students' ages with the scales. No statistical control for confounding variables was applied which limits causal inference.

## 3 Results

### 3.1 Sample Description

The mean age of the students included in the study was 21.77 ± 2.01, 78.1% were female, 21.9% were male, 57.5% were studying nursing, 42.5% were studying nutrition and dietetics, 40.9% were in their 2nd year, 33% were in their 3rd year, and 26.1% were in their 4th year. It was determined that 71.9% of the students graduated from Anatolian High School, 6.8% from Sciences/Imam Hatip High School, 21.3% from Health Vocational High School, 60.2% lived in the province, 25.1% in a district, 14.7% in a town/village, 62.1% perceived their family income level as equal to their expenses, 15.8% had their income more than their expenses, 22.1% had their income less than their expenses, 38.9% had 1–2 siblings, 33.4% had 3–4 siblings and 27.7% had more than 5 siblings (Table 1).

**TABLE 1:** Distribution of socio‐demographic characteristics of students (*n* = 570).

Variables	*n*	%
Age (Mean ± SD): 21.77 ± 2.01 (Min: 19—Max: 47)		
Gender		
Female	445	78.1
Male	125	21.9
Department of education		
Nursing	328	57.5
Nutrition and dietetics	242	42.5
Class		
2nd grade	233	40.9
3rd grade	188	33.0
4th grade	149	26.1
Graduated high school		
Anatolian High School	410	71.9
Science/Imam Hatip High School	39	6.8
Health Vocational High School	121	21.3
Place of residence		
Province	343	60.2
District	143	25.1
Town/Village	84	14.7
Perception of family income level		
Income is more than expense	90	15.8
Income equals expense	354	62.1
Income is lower than expenses	126	22.1
Number of siblings		
1–2	222	38.9
3–4	190	33.4
5 and above	158	27.7
Total	570	100.0

Abbreviation: SD, standard deviation.

### 3.2 Students' Opinions on Distance Education

When Table 2 is examined, 56.1% of the students have difficulty in continuing their education at the faculty with the distance education system, 52.5% have no difficulty in following the lessons due to limited opportunities in terms of internet and computers, 68.9% are concerned about the efficiency of the lessons with the distance education system and 58.1% do not find it sufficient to give theoretical lessons remotely. It was determined that 64.4% of the students have more difficulty studying with distance education than studying with face‐to‐face education, 59.1% do not have difficulty in accessing information sources (lecture notes, library etc.) with distance education and 73.3% do not have difficulty in using information technologies (Table 2).

**TABLE 2:** Distribution of students' opinions on the distance learning process (*n* = 570).

Variables	*n*	%
Difficulty in attending faculty through distance education or face‐to‐face education		
Distance education system	320	56.1
Face to face education system	250	43.9
Having difficulty following the lessons due to my limited internet and computer capabilities		
Yes	271	47.5
No	299	52.5
Concern about the efficiency of lessons with the distance education system		
Yes	393	68.9
No	177	31.1
The situation of thinking that giving theoretical courses remotely is sufficient		
Yes	239	41.9
No	331	58.1
The difficulty of studying via distance education compared with studying via face‐to‐face education		
Yes	367	64.4
No	203	35.6
Difficulty in accessing information resources (lecture notes, library, etc.) through distance education		
Yes	233	40.9
No	337	59.1
Difficulty in using information technologies		
Yes	152	26.7
No	418	73.3
Total	570	100.0

### 3.3 The Average Scores Students Received From the Scales

When the mean scores of the students participating in the study were examined, it was seen that the general fatigue sub‐dimension was 3.05 ± 1.16, the visual fatigue sub‐dimension was 2.93 ± 1.20, the social fatigue sub‐dimension was 2.73 ± 1.24, the emotional fatigue sub‐dimension was 2.78 ± 1.24, the motivational fatigue sub‐dimension was 2.82 ± 1.05 and the total scale was 2.86 ± 1.07. It was determined that they received points. It is observed that the students' general burnout and fatigue sub‐dimension scores are high, while the visual, emotional, motivational burnout and fatigue sub‐dimensions and the total scale score are at a moderate level. Scale scores were interpreted as low (1.00–2.33), medium (2.34–3.66) and high (3.67–5.00).

### 3.4 Comparing Students' Distance Learning Characteristics With Their Average Scores on Scales

Examining Table 3, it was found that there is a statistically significant difference between the mean scores of the Zoom Burnout and Fatigue Scale and its sub‐dimensions according to whether students have difficulties while attending the faculty with the distance education system or the face‐to‐face education system, have difficulties in following the courses due to limited possibilities in terms of internet and computers, worry about the efficiency of the courses with the distance education system, and think that the delivery of theoretical courses at a distance is sufficient. It can be seen that burnout and fatigue levels are higher among students who continue their faculty with the distance education system, have difficulties in following the courses due to limited facilities in terms of internet and computers, worry about the efficiency of the courses with the distance education system, and think that the delivery of theoretical courses at a distance is not sufficient (*p* < 0.001). When the effect sizes are examined, they are seen to be small, medium and large (Table 3).

**TABLE 3:** Zoom Burnout and Fatigue Scale and its sub‐dimensions according to students' opinions on the distance education process comparison of mean scores (*n* = 570).

Variables	General fatigue	Visual fatigue	Social fatigue	Emotional fatigue	Motivational fatigue	Total ZBFS
Difficulty in attending faculty through distance education or face‐to‐face education						
Distance education system	3.29 ± 1.08	3.12 ± 1.16	2.89 ± 1.20	2.98 ± 1.17	3.04 ± 0.96	3.07 ± 0.99
Face to face education system	2.74 ± 1.19	2.68 ± 1.22	2.53 ± 1.28	2.52 ± 1.28	2.53 ± 1.10	2.60 ± 1.12
*t*	5755	4411	3434	4475	5990	5249
*p*	*p* < 0.001	*p* < 0.001	*p* < 0.001	*p* < 0.001	*p* < 0.001	*p* < 0.001
Effect sizes (η^2^)	0.055	0.033	0.020	0.034	0.059	0.046
Having difficulty following the lessons due to my limited internet and computer capabilities						
Yes	3.40 ± 1.06	3.24 ± 1.15	3.11 ± 1.19	3.17 ± 1.18	3.16 ± 0.97	3.21 ± 1.01
No	2.74 ± 1.16	2.64 ± 1.19	2.39 ± 1.19	2.43 ± 1.19	2.51 ± 1.03	2.54 ± 1.03
*t*	7097	6054	7.233	7.435	7677	7860
*p*	*p* < 0.001	*p* < 0.001	*p* < 0.001	*p* < 0.001	*p* < 0.001	*p* < 0.001
Effect sizes (η^2^)	0.081	0.061	0.084	0.089	0.094	0.098
Concern about the efficiency of lessons with the distance education system						
Yes	3.40 ± 1.05	3.26 ± 1.13	3.01 ± 1.21	3.10 ± 1.18	3.10 ± 0.98	3.18 ± 0.99
No	2.28 ± 1.02	2.18 ± 1.02	2.13 ± 1.10	2.05 ± 1.06	2.18 ± 0.94	2.16 ± 0.92
*t*	11,943	10,901	11,339	8219	10,156	10,507
*p*	*p* < 0.001	*p* < 0.001	*p* < 0.001	*p* < 0.001	*p* < 0.001	*p* < 0.001
Effect sizes (η^2^)	0.201	0173	0.106	0.154	0.163	0.190
The situation of thinking that giving theoretical courses remotely is sufficient						
Yes	2.62 ± 1.15	2.54 ± 1.19	2.38 ± 1.22	2.38 ± 1.22	2.44 ± 1.06	2.47 ± 1.07
No	3.37 ± 1.07	3.21 ± 1.14	2.99 ± 1.20	3.07 ± 1.18	3.08 ± 0.96	3.14 ± 0.99
*t*	−8049	−6845	−6.003	−6834	−7.474	−7.773
*p*	*p* < 0.001	*p* < 0.001	*p* < 0.001	*p* < 0.001	*p* < 0.001	*p* < 0.001
Effect sizes (η^2^)	0.102	0.076	0.060	0.076	0.090	0.096
The difficulty of studying via distance education compared with studying via face‐to‐face education						
Yes	3.44 ± 1.04	3.28 ± 1.12	3.06 ± 1.18	2.14 ± 1.12	3.17 ± 0.95	3.23 ± 0.97
No	2.36 ± 1.05	2.28 ± 1.07	2.81 ± 1.19	2.06 ± 1.07	2.18 ± 0.92	2.30 ± 0.93
*t*	11,864	10,375	9094	11,308	12,037	12,211
*p*	*p* < 0.001	*p* < 0.001	*p* < 0.001	*p* < 0.001	*p* < 0.001	*p* < 0.001
Effect sizes (η^2^)	0.199	0.159	0.127	0.184	0.203	0.208
Difficulty in accessing information resources (lecture notes, library, etc.) through distance education						
Yes	3.48 ± 1.06	3.38 ± 1.10	3.21 ± 1.17	3.30 ± 1.17	3.27 ± 0.96	3.33 ± 0.99
No	2.76 ± 1.14	2.61 ± 1.18	2.41 ± 1.19	2.42 ± 1.16	2.50 ± 1.00	2.54 ± 1.01
*t*	7696	7882	7957	8909	9230	9.255
*p*	*p* < 0.001	*p* < 0.001	*p* < 0.001	*p* < 0.001	*p* < 0.001	*p* < 0.001
Effect sizes (η^2^)	0.094	0.099	0.100	0.123	0.130	0.131
Difficulty in using information technologies						
Yes	2.78 ± 1.26	2.96 ± 1.23	2.56 ± 1.26	2.61 ± 1.28	2.62 ± 1.07	2.71 ± 1.10
No	2.98 ± 1.16	3.07 ± 1.10	2.75 ± 1.27	2.78 ± 1.21	2.83 ± 1.02	2.88 ± 1.03
*t*	6645	5758	7591	7812	7670	7858
*p*	*p* < 0.001	*p* < 0.001	*p* < 0.001	*p* < 0.001	*p* < 0.001	*p* < 0.001
Effect sizes (η^2^)	0.072	0.055	0.092	0.097	0.094	0.098

Abbreviations: *t*, *t*‐test; ZBFS, Zoom Burnout and Fatigue Scale.

It was found that there was a significant difference between the mean scores of the Zoom Burnout and Fatigue Scale and its sub‐dimensions according to the students included in the study, the difficulty of studying in distance education compared with studying in face‐to‐face education, the difficulty of accessing information sources (lecture notes, library, etc.) in distance education, and the difficulty of using information technologies. It was observed that those who studied with distance education, those who had difficulty in accessing information sources (lecture notes, library, etc.) with distance education, and those who had difficulty in using information technologies had higher burnout and fatigue levels (*p* < 0.001). When the effect sizes are examined, they are seen to be small, medium and large (Table 3).

### 3.5 Results of Regression Analysis Regarding Factors Predicting the Zoom Burnout and Fatigue Scale Scores

Predicting students' overall experience levels in distance education shows that the model is statistically significant (*F* = 31.155; *p* < 0.001). The established model explains 28% of the variation in students' distance education experience (*R* ^2^ = 0.280).

According to the analysis results, the variables of difficulty in studying (β = −0.241), concern about academic efficiency (β = −0.157), limited internet and computer access (β = −0.113), and difficulty accessing information resources (β = −0.093) significantly predict the distance education experience negatively. This indicates that as students experience more difficulties in their study processes, concerns about educational efficiency, and limitations in accessing technical infrastructure, their overall experience with distance education weakens significantly. The fact that difficulty in studying is the strongest negative predictor shows that obstacles in academic process management play a decisive role in student experience. On the other hand, factors such as perceived difficulty between distance and face‐to‐face education, the adequacy of delivering theoretical courses remotely, and information technology skills were found to have no significant effect on the overall experience score (*p* > 0.05) (Table 4).

**TABLE 4:** Regression analysis of factors predicting the Zoom Burnout and Fatigue Scale scores.

Independent variables	β	SH	*t*	*p*	95% confidence interval (Lower–Upper)
Constant	4.639	0.305	15.217	0.000	4.040 to 5.237
Difficult situation	0.053	0.092	1.246	0.213	−0.066 to 0.295
Internet/Computer limitations	−0.113	0.093	−2.620	0.009*	−0.427 to −0.061
Concerns about lesson effectiveness	−0.157	0.125	−2.910	0.004*	−0.611 to −0.119
Theoretical course competency	0.072	0.096	1.631	0.103	−0.032 to 0.346
Study difficulty	−0.241	0.120	−4.500	0.000**	−0.775 to −0.304
Accessing information resources	−0.093	0.102	−1.980	**0.048** *	−0.403 to −0.002
Use of information technologies	−0.068	0.107	−1.548	0.122	−0.375 to 0.044

*Note:* *R* = 0.529; *R* ^2^ = 0.280; *F* = 31.155; Durbin–Watson = 1.851. Bold values indicates *p < 0.05; **p < 0.001.

## 4 Discussion

This research, which was conducted to determine the online burnout and fatigue states of students studying at the Faculty of Health Sciences of a university and the affecting factors, was discussed with the relevant literature.

The results indicate that students experienced moderate levels of the Zoom Burnout and Fatigue, suggesting that prolonged exposure to online education may negatively affect students' psychological well‐being, academic motivation and learning efficiency. Scale scores were interpreted as low (1.00–2.33), medium (2.34–3.66) and high (3.67–5.00). According to this classification, students' overall burnout and fatigue subscale scores were high, while their visual, emotional and motivational subscale scores, as well as their total scale score, were at a medium level. It was determined that students' scores on the general burnout and fatigue subscale were at a high level, while their scores on the visual, emotional and motivational burnout and fatigue subscales, as well as their total scale scores, were at a moderate level. To facilitate the interpretation of the scale scores, a classification of low, medium and high levels was made considering the scale's score ranges. Accordingly, students' general burnout and fatigue levels were in the high‐level category, while the other subscales and the total scale score were evaluated in the moderate‐level category. Although the burnout and fatigue levels were not severe, the results highlight that continuous participation in virtual learning environments may create emotional exhaustion and reduced engagement among students. In a study, dentistry students showed high levels of burnout syndrome (Montiel‐Company et al. 2016). In another study, it was determined that the quality of life of nurses working in hospitals decreased as their burnout increased during the pandemic. In addition, it can be said that the emotional exhaustion and depersonalization sub‐dimension of the Maslach Burnout Inventory and the compassion fatigue sub‐dimension of the Professional Quality of Life Scale explain the total variance (Doğu et al. 2024). In a study conducted with nursing students during the COVID‐19 process, the students were found to have high levels of burnout (Svavarsdottir et al. 2023). In a study conducted with nursing students, their academic burnout was found to be high (Chen et al. 2023). A study found that 54.7% of students in North America reported that their workload had increased and was significantly greater as a result of the transition to online learning, making fatigue and burnout more likely (Kakuchi 2021). Another study determined that academic burnout caused by students creates a reluctance to learn and causes fatigue (Xie et al. 2019). When studies are examined, it is seen that the burnout and fatigue levels of students studying in different academic units are related to each other. In this study, it is seen that students experience above‐average levels of burnout and fatigue during their online learning. When the study results are examined, it is seen that there are similar results to the findings of this research. The reason for this similarity is thought to be that the students have practical courses, cannot come to the practice areas due to the online learning method, and constantly learn via Zoom. It is of great importance that units providing education in the field of health sciences determine their policies for distance education and make the necessary plans. These results emphasize the importance of developing supportive and student‐centred online learning strategies to reduce the negative effects of prolonged screen exposure and digital learning fatigue.

The results of this study demonstrated that students who continued their education through the distance education system and experienced difficulties following courses due to limited access to internet and computer resources had higher levels of burnout and fatigue. This finding suggests that insufficient technological infrastructure and limited digital access may negatively affect students' ability to participate effectively in online learning environments. Difficulties in accessing educational materials and maintaining continuity in lessons may increase academic stress, emotional exhaustion and learning‐related fatigue among students. These findings emphasize the importance of improving technological support and ensuring equal access to digital resources in order to reduce the negative psychological effects associated with online education processes. Similar to this research finding, in a study conducted with senior students, more than half of the students stated that they had difficulty following the courses due to limited computer and internet opportunities or technical problems experienced on the platform where distance education is carried out. The attitudes of the students who had difficulties were found to be statistically significantly lower. It is thought that the limitations in the processing of application‐based subjects, especially in the field of health sciences, where theoretical knowledge is transformed into skills in applied courses, affect the efficiency of the courses (Hadımlı et al. 2023). In many studies conducted on online education, the technological problems experienced by students are stated as a disadvantage of online education (Birişçi 2013; Buluk and Esenti 2020; Öztürk and Özer 2022; Yilmaz et al. 2021). When the study results that are similar to these research findings are examined, it is striking that the studies were conducted in the same country and culture. In the pandemic and post‐earthquake period, the biggest fear of students is that they will not be able to provide adequate treatment and care to the patient if they start working life because they take practical courses online. In this case, students inevitably experience burnout and fatigue due to online education. Policies need to be adopted to adapt the curricula and training in the faculty of health sciences to emergencies such as pandemics and earthquakes. In Türkiye, distance education activities are generally conducted within universities. The Council of Higher Education (CHE), responsible for coordinating universities in Türkiye, also has authority in distance education activities. It exercises this authority through the Distance Education Board within CHE. After the pandemic, CHE published a document comprehensively addressing distance education under the new conditions. This document, titled ‘The New Normalization Process in the Global Pandemic’, discusses the changes that occurred in the higher education system during the pandemic. A noteworthy point in this policy document is that a ‘hybrid’ education model can be implemented even after the pandemic. It is seen that structuring and organization are important for achieving the desired results from the distance education model, and therefore, universities have important responsibilities. The document also addresses the psychological strain on individuals during the distance education process and recommends extracurricular online activities in this regard (YÖK 2020; Karasoy et al. 2021).

The results of this study indicate that students who attended courses through distance education experienced higher levels of burnout and fatigue. Rather than being attributable to online education alone, this finding likely reflects the cumulative effects of prolonged screen exposure, reduced face‐to‐face interaction, increased cognitive demands, and limited opportunities for social and academic engagement. These factors may collectively reduce students' psychological resources, making it more difficult to sustain attention, regulate emotions and maintain academic motivation over time. Consequently, burnout and fatigue should be considered multidimensional outcomes influenced by both the educational environment and the availability of technological and social support.

Previous studies support this interpretation. Çelikkanat et al. (2024) reported high levels of digital addiction among nursing students, suggesting that intensive technology use may have adverse psychological consequences when not accompanied by healthy digital habits. Similarly, Dolu et al. (2024) identified technical difficulties as one of the major barriers to effective distance education. Such barriers may not only interrupt the learning process but also increase frustration, cognitive load and academic stress, thereby contributing to burnout and fatigue. Kreijns et al. (2022) further emphasized that the physical separation inherent in online education reduces opportunities for meaningful interpersonal interaction, weakening students' sense of belonging and engagement. When communication barriers are combined with technological limitations, students may experience greater academic isolation, which has consistently been associated with poorer psychological well‐being.

The results should also be interpreted within the broader context of digital inequality. Although the COVID‐19 pandemic accelerated the expansion of distance education in Türkiye and increased access to digital learning opportunities (Babaoğlu and Kulaç 2021; Karasoy et al. 2021), the benefits of these developments have not been distributed equally. Access to reliable internet infrastructure, adequate technological devices and digital competencies remains uneven, particularly among students living in rural areas. Therefore, the effectiveness of distance education depends not only on the availability of online learning platforms but also on students' capacity to access and effectively utilize these resources.

An important contextual consideration in the present study is that nearly 40% of the participants resided in districts or villages. This characteristic may partially explain the higher burnout and fatigue levels observed, as students living in rural settings are more likely to encounter unstable internet connections, inadequate technological infrastructure and fewer educational resources. These challenges may increase the effort required to participate in online learning and consequently intensify psychological exhaustion. Therefore, geographical disparities should be regarded as a potential contributing factor when interpreting the relationship between distance education and student well‐being. Interestingly, the rapid digital transformation of higher education also suggests that familiarity with educational technologies may function as a protective factor. Students with stronger digital competencies may experience fewer technical difficulties, adapt more easily to virtual learning environments, and maintain more effective communication with peers and instructors. In contrast, students with limited digital literacy may perceive online learning as more demanding, thereby increasing their susceptibility to burnout and fatigue. This interpretation highlights that technological competence may moderate the impact of distance education on students' psychological outcomes. Overall, the present findings suggest that the adverse effects of distance education are not inevitable but depend largely on the quality of technological infrastructure, opportunities for meaningful social interaction, institutional support and students' digital competencies. Universities should therefore prioritize interactive instructional strategies, strengthen technical support services, improve digital accessibility and implement interventions that promote students' psychological well‐being. Future research should further investigate the mediating and moderating roles of digital literacy, socioeconomic status, internet accessibility and perceived social support in explaining the relationship between distance education and burnout and fatigue.

### 4.1 Limitations

This study was conducted only with 2nd‐, 3rd‐ and 4th‐year students from the Nursing and Nutrition and Dietetics Departments of a single university's Faculty of Health Sciences; therefore, the results cannot be generalized to all students. The data are limited by the scales used and the research group. Furthermore, the cross‐sectional design does not allow for the establishment of causal relationships. Data collection based on self‐reports increases the risk of social desirability bias and self‐choice bias. Inadequacies in internet infrastructure and the exclusion of other potential variables are also among the limitations of the findings. It was taken into consideration that the analyses were exploratory in nature and carried an increased risk of Type I errors. No statistical control for confounding variables was applied which limits causal inference.

### 4.2 Relevance to Clinical Practice

Since nursing is a hands‐on profession, practices must take place in a hospital setting and face‐to‐face. Online education should be used as a supplementary tool. Students should apply the online education they receive to their practice. Regular information meetings can be held for students at the beginning of each academic year regarding distance learning. Online participation exercises and training can be demonstrated to students in communication labs using role‐playing. Students can also be provided with training on the use of artificial intelligence.

It can be suggested that when delivering theoretical courses via distance education, active learning methods should be used, students' access to course materials should be facilitated and their participation in the courses should be activated. It is important for universities to update their policies regarding the distance education system and take the necessary precautions for emergencies. In addition, university educators need to create a well‐structured learning environment for students in order to improve student learning outcomes and reduce the impact of uncertainty, anxiety and stress on learning. It is recommended that studies be conducted to examine the distance education status of students. It is also recommended that university psychological counselling centers regularly provide training for students experiencing burnout and motivational fatigue.

## 5 Conclusion and Implications

The results of the study indicate that students experienced significant academic, technological and psychosocial difficulties during the distance education process. In particular, students who had difficulty attending faculty through the distance education system, had limited access to the internet and computers, were concerned about the efficiency of online courses, and considered the delivery of theoretical courses through distance education insufficient demonstrated higher levels of burnout and fatigue. In addition, students who experienced difficulties studying through distance education, had problems accessing information resources, and struggled to use information technologies were found to have higher levels of the Zoom Burnout and Fatigue.

In line with these results, it is important in nursing education to support students' digital learning competencies, improve access to technological resources, and develop psychosocial support programmes for online education processes. Furthermore, within the scope of evidence‐based nursing practices, it is recommended to implement regular break interventions aimed at reducing screen exposure, increasing the use of interactive teaching methods, and applying educational strategies that strengthen student engagement in digital learning environments. In this way, it may be possible to support students' academic performance, reduce burnout levels and ensure more sustainable learning processes.

## Author Contributions

**Papatya Karakurt:** conceptualization, investigation, writing – original draft, methodology, validation, visualization, writing – review and editing, project administration, formal analysis, software, data curation, supervision, resources. **Şeyda Yetginler Memiş:** conceptualization, funding acquisition, writing – original draft, visualization, validation, formal analysis, data curation, resources. **Meryem Firat:** conceptualization, investigation, funding acquisition, methodology, validation, writing – review and editing, formal analysis, resources.

## Funding

The authors have nothing to report.

## Ethics Statement

Research ethics approval was granted by the Erzincan Binali Yıldırım University Ethics Committee approval (29/12/2023 protocol no: 12/07) and written permission from the institution where the research was conducted. The online survey was distributed to student participants via social media groups after the course was completed and their grades were announced to limit any potential bias or coercion. Informed consent was obtained from the students prior to the survey questions. The online consent form outlined the aims of the study and emphasized how their anonymity, privacy and confidentiality would be protected, allowing students to decide whether to participate in the study.

## Conflicts of Interest

The authors declare no conflicts of interest.

## Data Availability

These copyrighted data are available from the corresponding author upon reasonable request.
